# Editorial: Toward and Beyond Human-Level AI

**DOI:** 10.3389/fnbot.2020.617446

**Published:** 2020-12-09

**Authors:** Witali Dunin-Barkowski

**Affiliations:** Department of Neuroinformatics, Center for Optical Neural Technologies, Scientific Research Institute for System Analysis, Russian Academy of Sciences, Moscow, Russia

**Keywords:** artificial intelligence, strong AI, learning to learn, sample-efficient learning, real time learning

Currently the agenda of human level Artificial Intelligence [also: “strong AI,” “Artificial General Intelligence (AGI),” etc.] is one of the most important problems of interest for the scientific community and the general public. However, due to many objective and subjective reasons (Bostrom, 2014), specific research and engineering works in this field are scarce. Below is a review of four papers that have been published in our Research Topic followed by a brief outline of the ultimate developments in AGI.

The first article of this Topic (Karimpanal and Bouffanais), focused on the problem of improved experience replay techniques in reinforcement learning algorithms. To learn more efficiently, the authors proposed an approach to select appropriate transition sequences to accelerate learning. The new method utilized a combination of tracking and storage, construction, and replaying of suitable transition sequences associated with higher magnitudes of temporal difference error. The approach was evaluated in an off-policy setting on such tasks as the puddle-world and mountain-car demonstrating a significant improvement in learning speed by controllable memory parameters.

The paper of Tapia et al. is connected with the ability of our brain to build and learn through observation of motor-motifs required for effective interaction with the environment. The authors developed a model for constructing behaviors in time-changing situations based on the semantic knowledge of actions using the dynamics of the neural network. Their results pointed to some form of static internal representation mechanism at the cognitive level involved in decision-making and strategy planning for the construction of a generalized map. Results of cognitive-motor skills were reported in tasks of a combat game of virtual fencing (defend and attack) and were validated experimentally using a real robot.

In a brief overview, Bac and Zinovyev described modern approaches to project multi-dimensional space on a lower-dimensional space designated by an analogy lizard brain task. Providing insight about the local intrinsic dimensionality based on the notions of mathematical projection theory, their review can be helpful for practical selection of methods which extract and detect a useful low-dimensional representation in AI applications. They list 100 references that show several injective, projective, and multiple manifold techniques characterized by measure similarity or dissimilarity. So, the development of new mathematical methods of data analysis is both now and in the near future one of the most important tasks for creating learning systems.

Lastly, Tyukin et al. developed a framework for the process of knowledge spreading across AI systems without requiring significant computational resources. They showed how AI systems produce a training environment for independent AI agents by using the pre-trained convolutional neural network. The authors used two learning algorithms to fully automate the passage of knowledge and experience in which one operates as a “teacher” and the other as a “student.” The framework was used for automated pedestrian detection in video streams and showed extreme selectivity for filtering false positive and false-negative errors.

The Research Topic showcases the diversity of its contents and impressive progress. Due to the dramatic events associated with the coronavirus pandemic, public interest for strong AI has decreased. Nevertheless, it is clear that either natural or artificial intelligence ought to be in strong demand to deal with the tough challenges of our currently stormy times. So, there remains an objective reason to get strong AI as soon as possible.

Certainly, it's difficult to predict the future of this field, but the era of strong AI is imminent and prospects for the development in several directions are suggested here. On the optimistic side, the problem of creating AI the human mind-level will mimic travel into a powerful technological “storm.” This view on the current state of AI has been recently reiterated by the founder of reinforcement learning Rich Sutton (Sutton, 2019). Additionally, this idea is confirmed in a recent pre-print which is submitted by the OpenAI team (Brown et al., 2020). Thus, this research direction has demonstrated substantial gains on many natural language processing tasks and benchmarks.

A second direction is combining AI applications with the development of newly discovered powerful mathematical tools. This approach has been pursued by Gorban (1988) still shows immanent progress (Tyukin et al.).

A third direction has been presented recently by Lillicrap et al. (2020). The main idea here is to trace down real neural analogs of error backpropagation, long thought to be possible only in artificial neural systems. Successful deciphering of those mechanisms might help substantially accelerate AI learning rates.

A final direction, with the concept of goal-directed training, is proposed to use a custom assemblage of existing AI components in an autonomous intelligent construction, in which knowledge should be laid in real-time by human teachers and operators of the system (Dunin-Barkowski and Shakirov, 2019). It is supposed that strong AI will emerge in the technical system as a result of a permanently human-guided teaching/learning process.

In summary, the prospects for solving the problem of strong AI remain uncertain but we still have the chance to achieve that goal soon.

## Author Contributions

The author confirms being the sole contributor of this work and has approved it for publication.

## Conflict of Interest

The author declares that the research was conducted in the absence of any commercial or financial relationships that could be construed as a potential conflict of interest.
